# An improved preparation of phorbol from croton oil

**DOI:** 10.3762/bjoc.13.133

**Published:** 2017-07-11

**Authors:** Alberto Pagani, Simone Gaeta, Andrei I Savchenko, Craig M Williams, Giovanni Appendino

**Affiliations:** 1 Dipartimento di Scienze del Farmaco, Università degli Studi del Piemonte Orientale, Largo Donegani 2, 28100 Novara, Italy; 2 School of Chemistry and Molecular Biosciences, University of Queensland, 4072, Brisbane, Australia

**Keywords:** croton oil, diterpenoids, natural products, phorbol, transesterification

## Abstract

**Background:** Croton oil is the only commercial source of the diterpenoid phorbol (**1a**), the starting material for the semi-synthesis of various diesters extensively used in biomedical research to investigate cell function and to evaluate in vivo anti-inflammatory activity. While efficient chemoselective esterification protocols have been developed for phorbol, its isolation from croton oil is technically complicated, and involves extensive manipulation of very toxic materials like the oil or its native diterpenoid fraction.

**Results:** The preparation of a crude non-irritant phorboid mixture from croton oil was telescoped to only five operational steps, and phorbol could then be purified by gravity column chromatography and crystallization. Evidence is provided that two distinct phorboid chemotypes of croton oil exist, differing in the relative proportion of type-A and type-B esters and showing different stability to deacylation.

**Conclusion:** The isolation of phorbol from croton oil is dangerous because of the toxic properties of the oil, poorly reproducible because of differences in its phorboid profile, and time-consuming because of the capricious final crystallization step. A solution for these issues is provided, suggesting that the poor-reproducibility of croton oil-based anti-inflammatory assays are the result of poor quality and/or inconsistent composition of croton oil.

## Introduction

Croton oil is obtained by pressing or solvent extraction from the seeds of *Croton tiglium* L., a small tree native to the Far East [1]. The oil is toxic to all living organisms, from bacteria to insects and vertebrates, and its irritancy and cathartic properties are legendary [2]. Croton oil was once used in human medicine as a topical rubefacient and in veterinarian medicine as a strong laxative [1], but nowadays its only medical use is in rejuvenating esthetic surgery in association to blepharoplasty, a practice that was mastered in the 1960s capitalizing on the potent exfoliating activity of the oil, especially in association to phenol [3].

**Figure 1:**
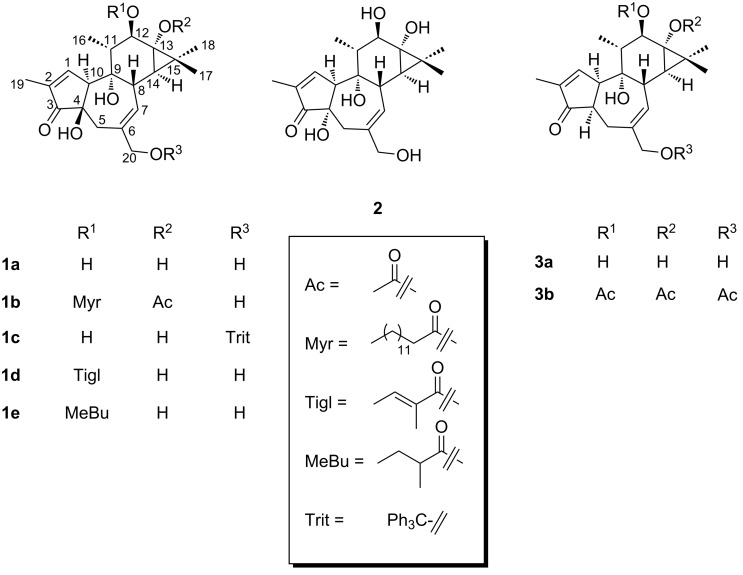
The major diterpene polyols from croton oil [phorbol (**1a**), 4α-phorbol (**2**), 4-deoxy-4α-phorbol (**3a**)] and their derivatives.

The extraordinarily obnoxious and vesicant properties of croton oil have fostered studies aimed at the identification of its active principles since the very early developments of organic chemistry. Thus, the first chemical study on croton oil was reported by Pelletier and Caventou, the founding fathers of alkaloid chemistry, in 1818 [4], but the nature of its irritant principles remained obscure and controversial until 1930, when Flaschenträger unambiguously characterized the inflammatory fraction of the oil as a mixture of esters of a crystalline diterpene pentaol, named phorbol (Figure 1, **1a**) after the plant family to whom *C. tiglium* belongs (Euphorbiaceae) [5]. The early studies left their mark in organic chemistry in the well-known names of crotonic and tiglic acids, although, paradoxically, croton oil does not contain crotonic acid, that is only generated in the harsh conditions of the early studies [6]. The structure of phorbol eluded clarification until 1968, when it was eventually elucidated by a low-temperature (−160 °C) crystallographic study on the chloroform solvate of its 20-(5-bromofuroate) [7]. This study solved a riddle that classic degradative studies had proved unable to address because of the tendency of phorbol to skeletal rearrangement and to its idiosyncratic chemical reactivity [6]. By this time, the medicinal use of croton oil had become obsolete, but interest had been rekindled by the discovery of its co-carcinogenic properties by Berenblum in 1941 [8]. The tumor-promoting properties of the oil were associated to a specific class of phorbol diesters, exemplified by phorbol myristate acetate (PMA, TPA, **1b**), having a long-chain and a short-chain acyl residue on the vicinal hydroxy groups on ring C (Type-A esters). The molecular target of PMA was identified in a series of isoforms of PKC, a family of serine/threonine kinases involved in a host of cellular activities [9]. Because of its kinase-activating properties, PMA has become an indispensable tool in the study of cell function, with a single vendor claiming to have sold over 250,000 ampules of TPA since 1980 [10]. PMA has also been clinically investigated as an anti-cancer agent [11], and, in the wake of the successful development of ingenol mebutate for the management of actinic keratosis, a pre-cancerous condition [12], interest for phorboids in the area of cancer prevention and treatment remains high [13].

Phorbol occurs in croton oil as a mixture of di- and triesters, generally in a ca. 1:2 ratio [6], and therefore isolation involves a deacylation step, critical because of the sensitivity of phorbol to isomerization to 4α-phorbol (**2**) by a base-induced vinylogous retro-aldol mechanism [6]. Furthermore, phorbol strongly retains all kinds of solvents, forming crystalline solvates of limited stability with many common solvents, including ethanol [6,10]. Over the past three decades, we have been interested in the chemistry of phorbol, and have practiced its preparation from croton oil, evaluating various strategies to minimize the contact with this very toxic and irritant material, simplifying the purification strategy, and improving the storage of the final product. After summarizing the methods previously described in the literature, we describe in detail the protocol we have developed and its modulation according to differences in the composition of commercial croton oil.

## Results and Discussion

The process developed by Flaschenträger [5] and then basically used by Hecker [6] and Crombie [14] in their classic studies on the structure of phorbol is based on the repeated extraction of croton oil with methanol to separate phorbol esters from triglycerides, and on the use of barium hydroxide as a base for the hydrolysis, using long (4–5 days) reaction times under mild basic conditions (pH ca. 8–9). After partition between ether and water, and precipitation of barium as a sulfate, the water phase is exhaustively extracted with ether and ethyl acetate, and then evaporated. Phorbol is extracted from the residue with hot methanol and, after filtration of the inorganic salts and evaporation, the methanol extract is crystallized from ethanol. There are two major issues with this protocol. The first one is the repeated handling of croton oil, a very toxic and obnoxious material, during the methanol extraction step, that requires several hours of stirring and then of resting to achieve a good phase separation, and the second one is the capricious crystallization of phorbol from the transesterification reaction mixture, a viscous oil for the presence of glycerol, that requires extended periods (four weeks according to ref. [6]). Furthermore, the unstable ethanol solvate has to be transformed into a more stable hydrate by recrystallization from hot water, a step that also requires a long time (one week according to ref. [6]). Also of concern is the overall number of operational steps (a series of over fifteen partitions, separations and evaporations), while the recovery of the more lipophilic and abundant phorbol triesters by methanol extraction of the oil is problematic, since these compounds are strongly retained in the triglyceride phase and multiple extractions are necessary to transfer them into the methanol phase, with an estimated loss of ca 50% [15]. To cope with the difficulties of the crystallization steps, protocols based on the purification of a crude mixture of ethanol solvates of phorboids by reverse-phase preparative flash chromatography or by countercurrent chromatography were developed [15–17]. The recovery of the triesters could be improved by first treating croton oil with acidic methanol to selectively remove the 20-acyl group. Partition between methanol and hexane afforded a crude mixture of phorbol diesters, that was next tritylated. After deacylation by basic treatment and chromatography, 20-tritylphorbol (**1c**) was obtained, as a hydrate, in sufficient purity to be used as starting material for the esterification [18]. This method was originally developed by Bernd Sorg of the Deutsches Krebsforschung Zentrum (Heidelberg, Germany), who kindly shared it with other researchers in the field. In our hands, it was simpler and more efficient than the original process developed by Flaschenträger, but it was, nevertheless, problematic for the multigram isolation of phorbol due to the extensive handling of the toxic croton oil and the ultra-toxic mixture of phorbol diesters obtained in the acidic transesterification step. Furthermore, the tritylation of the crude mixture of phorbol diesters suffered from interference from variable amounts of glyceryl mono- and diesters co-extracted with phorbol diesters and, presumably, already occurring in the native oil.

Since phorbol and its monoesters are not toxic, an “anticipation” of the deacylation step could afford a reaction mixture amenable to handling under normal laboratory conditions, also securing the recovery of phorbol triesters, difficult to selectively extract from the oil with a polar solvent. This strategy is not new in conception [16,19], but its implementation needs improvement in the recovery of phorbol to be practical. Thus, the water solution of phorbol and glycerol obtained after hydrolysis of the oil and washing with organic solvents must be evaporated with care, maintaining an acidic pH to minimize epimerization to 4α-phorbol [19]. Next, the recovery of phorbol from the resulting ca 10% solution of phorboids in glycerol is difficult under both normal- and reverse-phase silica gel chromatography, that fail to separate phorbol from 4α-4-deoxyphorbol (**3a**) because of their similar chromatographic behavior and of the presence of glycerol. As a result, the week-long crystallization from water was still necessary [19], a process that, tediousness aside, we found was accompanied by partial epimerization to 4α-phorbol (**2a**) and erosion of the overall final yield.

To streamline the recovery of phorbol in the deacylation step, croton oil was treated with sodium methylate. By replacing hydrolysis with transesterification, it was possible to remove fats from the detoxified reaction mixture by extraction with petroleum ether, making dilution with water unnecessary. Provided that the pH of the reaction did not exceeded 13, retro-aldol epimerization was also negligible, and undetectable by TLC control. Evaporation of methanol was straightforward and gave a solution of phorboids in glycerol as a dark thick oil that was subjected to liquid–liquid partition to recover phorbol from the glyceryl matrix. After considerable experimentation, we found that glycerol could be efficiently removed from a tetrahydrofuran (THF) solution of the transesterification residue by repeated washings with acidified brine. 1-Butanol and 1,4-dioxane were far less selective, affording extracts heavily contaminated by glycerol, also giving problems of foaming during their evaporation. The rationale for the selective partition between THF, a low-boiling and easily removed solvent, and water is unclear. The use of THF was inspired by the work of Seebach on the solubility of peptides in ether-type organic solvents in the presence of certain alkaline cations [20], and it is not unconceivable that the interaction with sodium ions substantially diversifies the relative polarity of glycerol and phorbol, making it possible to selectively partition them. In this way, the preparation of the diterpene polyol fraction was telescoped to only five operational steps (treatment of croton oil with sodium methylate, extraction with petroleum ether, evaporation, partition between THF and brine, and evaporation of THF). Purification of phorbol from the THF extract by gravity column chromatography (GCC) was then straightforward, affording a semi-crystalline ca. 5:1 mixture of phorbol (**1a**) and 4α-4-deoxyphorbol (**3a**). The two compounds had very similar chromatographic behavior, but could be efficiently separated by exploiting the efficient solubility of **3a** in ethyl acetate, a solvent where phorbol is insoluble. Thus, after trituration with ethyl acetate, filtration, and washing, phorbol could be obtained as an off-white powder (6.0 g, 1.2% from croton oil), sufficiently pure for further chemical modification and devoid of **3a**. The ethyl acetate solvate of phorbol is a powder with limited stability (weeks) also at low temperature, but crystallization from methanol afforded large crystals of the more stable methanol solvate. While the ethanol solvate of phorbol degrades in a few days even at low temperature [21], the methanol solvate could be stored for at least four months at 4 °C without any significant degradation.

Transesterification with sodium methylate, and presumably also with barium hydroxide under the milder conditions of the Flaschenträger protocol, could not remove the α-branched acyl group of type-B phorbol esters, and a mixture of phorbol 12-monoesters, mainly phorbol 12-tiglate (**1d**) and phorbol 12-(2-methylbutyrate) (**1e**) was obtained from the early chromatographic fractions. The mixture of 12-acyl phorbols that had resisted global transesterification could not be further hydrolyzed without epimerization to 4α-phorbol (**2**) and extensive degradation, and accounted for ca 30% of the amount of phorbol obtained from the transesterification. The recovery of phorbol from the monoesters **1d** and **1e** was, however, possible after tritylation of the primary 20-hydroxy group (vide infra).

While this method worked well with different batches of croton oil, with consistent yields of phorbol as EtOAc solvate in the range of 1%, some samples gave a lower yield (0.2–0.3%) because of incomplete transesterification. Furthermore, the crude transesterification mixture was devoid of significant amounts of 4α-4-deoxyphorbol (**3a**), and contained as major constituent a mixture of partially hydrolyzed esters, mainly phorbol 12-tiglate (**1d**) and phorbol 12-(2-methyl)butyrate (**1e**). Thus, the ^1^H NMR spectrum (methanol-*d*_4_) of the crude phorboid fraction, while showing the deshielded signals of the tiglate methine (δ ca. 6.80) and of H-1 of phorbol at δ ca. 7.60, lacked the singlet of H-1 of 4α-4-deoxyphorbol at δ ca. 7.20. Hydrolysis of the 12-monoesters failed under a variety of conditions, including hydrazinolysis for the tiglate residue, and required conditions too basic for the survival of phorbol. On the other hand, tritylation of the primary 20-hydroxy group had a surprising stabilizing effect toward basic degradation, making it possible to remove the remaining ester group. 20-Tritylphorbol (**1c**) [18] obtained in this way could be directly used for the synthesis of specific esters, or, alternatively, deprotected with acidic methanol (pH 3) to phorbol. The reasons for this trityl-induced stabilization are unclear, an educated guess being that the bulky trityl group could hinder oxidative reactions based on oxygen attack to the ring B double bond, a major degradation pathway for phorbol derivatives, especially under basic conditions [22].

Taken together, these observations revealed that two chemotypes of croton oil exist. The high-yielding oil contains mainly type-A phorbol di- and triesters. These phorboids have a long-chain acyl group bound to the secondary hydroxy group at C-12 and a short chain acyl group bound to the 13-hydroxy group, and are easily transesterified to phorbol. Conversely, the low-yield chemotype is dominated by type-B phorbol esters, where the long-chain ester group is located at the tertiary 13-hydroxy group, and branched acyl groups are bound to the 12-hydroxy. These branched acyl groups are not significantly removed by transesterification in the pH range of stability of phorbol, even at the more basic conditions of our protocol compared to the classic Flaschenträger method. Furthermore, 4α-4-deoxyphorbol derivatives are not contained in significant amounts in this chemotype.

Croton oil is used as a reference for in vivo anti-inflammatory assays, like the mouse-ear erythema assay, and it is tempting to suggest that the notoriously poor-reproducibility of the data from this assay [23] might be related also to differences in the composition of croton oil, since the irritancy of phorbol esters is critically dependent on their acylation profile [6]. However, the native phorboid profile of croton oil is still poorly characterized in terms of analytical profile [24], and the recovery of the native highly lipophilic phorboid esters from the lipid matrix of the oil remains a challenge. We hope that our observations will foster studies aimed at developing analytical methods to better characterize and quantify the diterpenoid profile of this oil, whose extraordinarily irritant properties have not only generated scientific interest, but also found a place in history [25] and literature [26].

## Experimental

**General experimental procedures:** ^1^H and ^13^C NMR spectra for the mixtures **1d**/**1e** were measured on a Bruker 700 Anance III HD (700.43 MHz; 176.13 MHz). Chemical shifts were referenced to the residual solvent signal (CDCl_3_: δH = 7.24, δC = 77.0). Silica gel 60 (70–230 mesh) for gravity column chromatography (GCC) was purchased from Macherey-Nagel (Düren, Gerrmay). Aluminum-coated Merck 60 F254 (0.25 mm) plates were used for TLC, visualizing the spots by UV inspection and/or staining with 5% H_2_SO_4_ in ethanol and heating. All solvents were of analytical grade, and were purchased from Aldrich, while croton oil was supplied by Adipogen Life Sciences (San Diego, USA). References samples of the batches used in this study are kept at the Novara laboratories.

### Phorbol from croton oil

**a) Croton oil rich of type-A esters:** In a 2 L round-bottom flask, freshly prepared 0.3 N sodium methylate in methanol was added dropwise (ca. 10 mL/min) to a magnetically stirred mixture of croton oil (LKT Laboratories, batch number 2597837, 500 mL) and methanol (50 mL) until the pH reached a value of 12–12.5 (pH strips, 0.5 pH unit resolution). Approximatively 1 L of methylate solution was necessary to reach and stabilize this pH value, and during the addition the amber color of the oil initially faded, and next darkened to eventually become black when the pH was strongly basic (>10). The course of the transesterification was followed by TLC, monitoring the appearance of the spot of phorbol [(EtOAc/MeOH 96:4 as eluent, direct deposition from the reaction mixture, *R*_f_ (phorbol) = 0.14)], and the lack of formation of 4α-phorbol (*R*_f_ = 0.09 in the same eluent system). After stirring overnight, the reaction mixture, whose pH was now around 11.5, was transferred into a 3 L separatory funnel, neutralized with a few drops of glacial acetic acid, and extracted with petroleum ether (5 × 300 mL). The upper phase was initially deep yellow, but its color faded with the successive extractions, while the lower phase remained dark colored. Evaporation of the lower methanol phase gave a viscous black residue that was dissolved in THF (250 mL) and washed with brine until the lower water phase was almost colorless (5 × 250 mL). The combined water phases were back-extracted with THF (ca. 100 mL), and the pooled THF phases were dried with sodium sulfate and then evaporated. A semi-solid residue was obtained (ca. 30 g), then purified by GCC on silica gel (250 g). The column was packed with petroleum ether/EtOAc 5:5 and the amount of EtOAc was gradually increased. Elution with petroleum ether/EtOAc 2:8 gave a crude fraction of phorbol monoester (8.9 g, see infra or the characterization). Elution was next continued with EtOAc and finally with EtOAc/MeOH 9:1 to afford a mixture of phorbol (**1a**) and 4α-4-deoxyphorbol (**3a**) as a semi-solid orange paste (ca. 15 g). The paste was triturated with EtOAc (150 mL) and the suspension was cooled two hours at the refrigerator temperature and next suction filtered to obtain phorbol as a slightly oatmeal-colored powder (6.0 g, 1.2% from the oil). Recrystallized from hot MeOH (35 mL) afforded 1.07 g of large colorless crystals of a methanol solvate.

The mother liquors from the trituration with EtOAc were evaporated, dissolved in pyridine (20 mL), and then treated with Ac_2_O (20 mL) and DMAP (cat.). After 1 h, the reaction was worked up by the addition of a few drops of methanol to destroy the excess Ac_2_O and of 2 N H_2_SO_4_, and next extracted with EtOAc. After drying and evaporation, the residue was purified by GCC on silica gel using petroleum ether/EtOAc 8:2 as eluent to afford 12,13,20-triiacetyl-4α-4-deoxyphorbol (**3b**, 1 g) [27] and 12,13,20-triacetylphorbol (500 mg) [27].

**b) Croton oil rich of type-B esters:** The oil (Alexis Biochemicals, batch number 350-089-0000, 500 mL) was processed as above. The de-glycerinated THF crude phorboid mixture was separated by GCC to afford 9.0 g of a mixture of phorbol monoesters and 1.0 g crude phorbol, that, when analyzed by ^1^H NMR was devoid of 4α-4-deoxyphorbol. A portion (1.0) of the phorbol monoesters mixture was further purified by GCC to obtain an orange powder, that was then washed with ether to afford a colorless product (400 mg). This, when analyzed by ^1^H NMR, was a mixture of the phorbol monoesters **1d** and **1e** (ca. 2:1 ratio).

**Phorbol-12-tiglate (1d):** ^1^H NMR (700.43 MHz, CDCl_3_) δ 7.56 (s, 1H, H-1), 5.63 (m, 1H, H-7), 4.84 (d, *J* = 9.8 Hz, 1H, H-12), 4.03 (m, 1H, H-20b), 3.98 (m, 1H, H-20a), 3.17 (br d, *J* = 2.5 Hz, 1H, H-10), 3.09 (m, 1H, H-8), 2.54 (m, 1H, H-5b), 2.44 (m, 1H, H-5a), 1.77 (dd, *J* = 2.9,1.3 Hz, 3H, H-19), 2.15 (m, 1H, H-11), 1.01 (s, 3H, H-17), 1.16 (s, 3H, H-16), 1.03 (s, 3H, H-18), 0.90 (m, 1H, H-14), 6.85 (qd, *J* = 7.1, 1.3 Hz, 1H, H-3’), 1.78 (m, 3H, H-4’), 1.80 (d, *J* = 1.09 Hz, 1H, H-5’), 4.93 (br s, 1H, 9-OH); ^13^C NMR (176.13 MHz, CDCl_3_) δ 208.80 (s, C-3), 160.28 (s, C-1), 140.74 (s, C-6), 133.41 (s, C-2), 129.29 (d, C-7), 79.17 (s, C-9), 87.36 (d, C-12), 73.49 (s, C-4), 67.97 (t, C-20), 60.83 (s, C-13), 56.77 (d, C-10), 43.50 (d, C-11), 38.99 (d, C-8), 38.74 (t, C-5), 35.19 (d, C-14), 27.69 (s, C-15), 22.27 (q, C-16), 17.05 (q, C-17), 16.08 (q, C-18), 10.14 (q, C-19), 170.51 (s, C-1’), 138.66 (s, C-3’), 128.13 (s, C-2’), 14.51 (q, C-4’), 12.05 (q, C-5’).

**Phorbol-12-(2-methylbutyrate)** (**1e**): ^1^H NMR (700.43 MHz, CDCl_3_) δ 7.55 (br s, 1H, H-1), 5.63 (br d, *J* = 5.1 Hz, 1H, H-7), 4.83 (d, *J* = 10.0 Hz, 1H, H-12), 4.03 (d, *J* = 12.9 Hz, 1H, H-20b), 3.98 (d, *J* = 12.9 Hz, 1H, H-20a), 3.16 (br d, *J* = 3.3 Hz, 1H, H-10a), 3.09 (br d, *J* = 5.6 Hz, 1H, H-8a), 2.54 (d, *J* = 19.1 Hz, 1H, H-5b), 2.44 (d, br, *J* = 16.3 Hz, 1H, H-5a), 1.80 (dd, *J* = 2.9, 1.3 Hz, 3H, H-19), 2.11 (dd, *J* = 10.0, 6.72 Hz, 1H, H-11), 1.04 (s, 3H, H-17), 1.17 (s, 3H, H-16), 1.00 (d, *J* = 6.5 Hz, 3H, H-18), 0.89 (d, *J* = 7.6 Hz, 1H, H-14a), 2.39 (td, *J* = 13.9, 7.0 Hz, 1H, H-2’), 1.65 (m, 1H, H-3’), 1.48 (tt, *J* = 13.7, 7.4 Hz, 1H, H-3’), 1.13 (d, *J* = 6.9 Hz, 1H, H-5’), 0.90 (t, *J* = 6.0 Hz, 3H, H-4’); ^13^C NMR (176.13 MHz, CDCl_3_) δ 208.77 (s, C-3), 160.18 (d, C-1), 140.84 (s, C-6), 133.42 (s, C-2), 129.17 (d, C-7), 79.12 (s, C-9), 87.41 (d, C-12), 73.47 (s, C-4), 67.92 (t, C-20), 60.89 (s, C-13), 56.82 (d, C-10), 43.28 (d, C-11), 38.96 (d, C-8), 38.70 (t, C-5), 35.14 (d, C-14), 27.63 (s, C-15), 22.37 (q, C-16), 17.24 (q, C-17), 15.92 (q, C-18), 10.13 (q, C-19), 179.68 (s, C-1’), 41.31 (d, C-2’), 26.90 (t, C-3’), 11. 67 (q, C-4’), 16.47 (q, C-5’).

**Hydrolysis of the mixture of 1d/1e:** A portion of the mixture of monoesters (3.0 g) was dissolved in pyridine (30 mL) and treated with trityl chloride (11.4 g) and cat. DMAP. After stirring overnight at room temp., the reaction was worked up by dilution with EtOAc (50 mL) and washing with 2 N H_2_SO_4_/brine (10:1, 100 mL). After drying and evaporation, the residue was purified by GCC on silica gel (petroleum ether/EtOAc 4:6 as eluent) to afford 3.14 g of a mixture of 20-tritylphorbol monoesters. The latter was dissolved in methanol, and 0.3 N sodium methylate was added dropwise until pH reached 12.5. After stirring overnight at room temp., the reaction was worked up by neutralization with 2 N H_2_SO_4_, dilution with brine, and extraction with CH_2_Cl_2_. Evaporation of the solvent left a solid residue, that was purified by GCC on silica gel (75 g, petroleum ether/EtOAc 3:7 as eluent) to afford 930 mg of 20-trityl phorbol. The latter could be directly used for the preparation of specific 12,13-diesters. Alternatively, it was dissolved in methanol (15 mL) and acidified to pH 3 with a few drops of 70% HClO_4_. After 30 min, the reaction was worked up by neutralization with NaOAc and evaporation. The residue was purified by GCC on silica gel (15 g) using EtOAc/MeOH 95:5 as eluent, to afford 460 mg phorbol, that was triturated with EtOAc, eventually affording 295 mg of a colorless powder.

## Supporting Information

File 1 ESI-HRMS and ^1^H and ^13^C NMR spectra of compounds **1d** and **1e**.
