# Supplementary material for: An improved preparation of phorbol from croton oil
Source: Beilstein J Org Chem. 2017 Jul 11;13:1361–7. doi: 10.3762/bjoc.13.133 (PMC5530722; doi:10.3762/bjoc.13.133)

Supporting Information

for

An improved preparation of phorbol from croton oil

Alberto Pagani[1], Simone Gaeta[1], Andrei I. Savchenko[2], Craig M. Williams[2] and Giovanni Appendino[*1]

Address: [1]Dipartimento di Scienze del Farmaco, Università degli Studi del Piemonte Orientale, Largo Donegani 2, 28100 Novara, Italy and [2]School of Chemistry and Molecular Biosciences, University of Queensland, 4072, Brisbane, Australia

Email: Giovanni Appendino - giovanni.appendino@uniupo.it
*Corresponding author

ESI-HRMS and ^{1}H and ^{13}C NMR spectra of compounds 1d and 1e

Table of Contents:

##	Title	page
1.	General Experimental Procedures.	2
2.	ESI HRMS of the sample **1d**/**1e** (2/1) in negative mode.	3
3.	ESI HRMS of the sample **1d**/**1e** (2/1) positive mode.	4
4.	Tabulated NMR data for **1d** compound recorded in $CDCl_3$ at 176/700 MHz.	5
5.	^{1}H NMR spectrum and assignments for **1d** recorded in $CDCl_3$.	6
6.	^{13}C NMR spectrum and assignments for **1d** recorded in $CDCl_3$.	6
7.	DEPT spectrum and assignments for **1d** recorded in $CDCl_3$.	7
8.	COSY spectrum for **1d** recorded in $CDCl_3$.	7
9.	HSQC edit spectrum for **1d** recorded in $CDCl_3$.	8
10.	HSQC spectrum for **1d** recorded in $CDCl_3$, 0.7-2.0×5-45 ppm expansion.	8
11.	HMBC spectrum for **1d** recorded in $CDCl_3$.	9
12.	HMBC spectrum for **1d** recorded in $CDCl_3$, 0.8-1.3×5-98 ppm expansion	9
13.	NOESY spectrum for **1d** recorded in $CDCl_3$	10
14.	Tabulated NMR data for **1e** compound recorded in $CDCl_3$ at 176/700 MHz.	11
15.	^{1}H NMR spectrum and assignments for **1e** recorded in $CDCl_3$.	12
16.	^{1}H NMR spectrum expansions for **1e** recorded in $CDCl_3$.	12
17.	^{13}C NMR spectrum and assignments for **1e** recorded in $CDCl_3$.	13
18.	^{13}C NMR spectrum expansions for **1e** recorded in $CDCl_3$.	13
19.	DEPT spectrum and assignments for **1e** recorded in $CDCl_3$,	14
20.	COSY spectrum for **1e** recorded in $CDCl_3$.	14
21.	HSQC edit spectrum for **1e** recorded in $CDCl_3$.	15
22.	HSQC spectrum for **1e** recorded in $CDCl_3$, 0.7-3.1×5-58 ppm expansion.	15
23.	HSQC edit spectrum for **1e** recorded in $CDCl_3$.	16
24.	HMBC spectrum for **1e** recorded in $CDCl_3$, 0.7-2.6×5-95 ppm expansion	16
25.	NOESY spectrum for **1e** recorded in $CDCl_3$.	17
26.	NOESY spectrum for **1e** recorded in $CDCl_3$, 0.5-5.0×0.7-3.3 ppm expansion	17

1. **General Experimental Procedures**. 1D and 2D NMR spectra were recorded on a Brucker 700 Avance III HD (^1H NMR - 700.47 MHz; ^{13}C NMR 176.13 MHz) in $CDCl_3$: δ in ppm, J in Hz. Low resolution electrospray ionisation mass spectrometry measurements (LRESIMS) were recorded in positive or negative ionization mode on a Bruker Esquire HCT (High Capacity 3D ion trap) instrument with a Bruker ESI source. High resolution electrospray ionisation (HRESIMS) accurate mass measurements were recorded in positive mode on a Bruker MicrOTOF-Q (quadrupole – Time of Flight) instrument with a Bruker ESI source. Accurate mass measurements were carried out with external calibration using sodium formate as reference calibrant.

2. ESI HRMS of the sample **1d**/**1e** (2/1) in negative mode.

Analysis Info

Analysis Name	D:\Data\Williams\APM69-neg.d	Acquisition Date	9/8/2016 4:49:05 PM
Method	tune_wide_pos_June 2016.m	Operator	BDAL@DE
Sample Name		Instrument	micrOTOF-Q 228888.00070
Comment			

Acquisition Parameter

Source Type	ESI	Ion Polarity	Negative	Set Nebulizer	0.4 Bar
Focus	Not active	Set Capillary	4000 V	Set Dry Heater	180 °C
Scan Begin	50 m/z	Set End Plate Offset	-500 V	Set Dry Gas	4.0 l/min
Scan End	3500 m/z	Set Collision Cell RF	550.0 Vpp	Set Divert Valve	Source

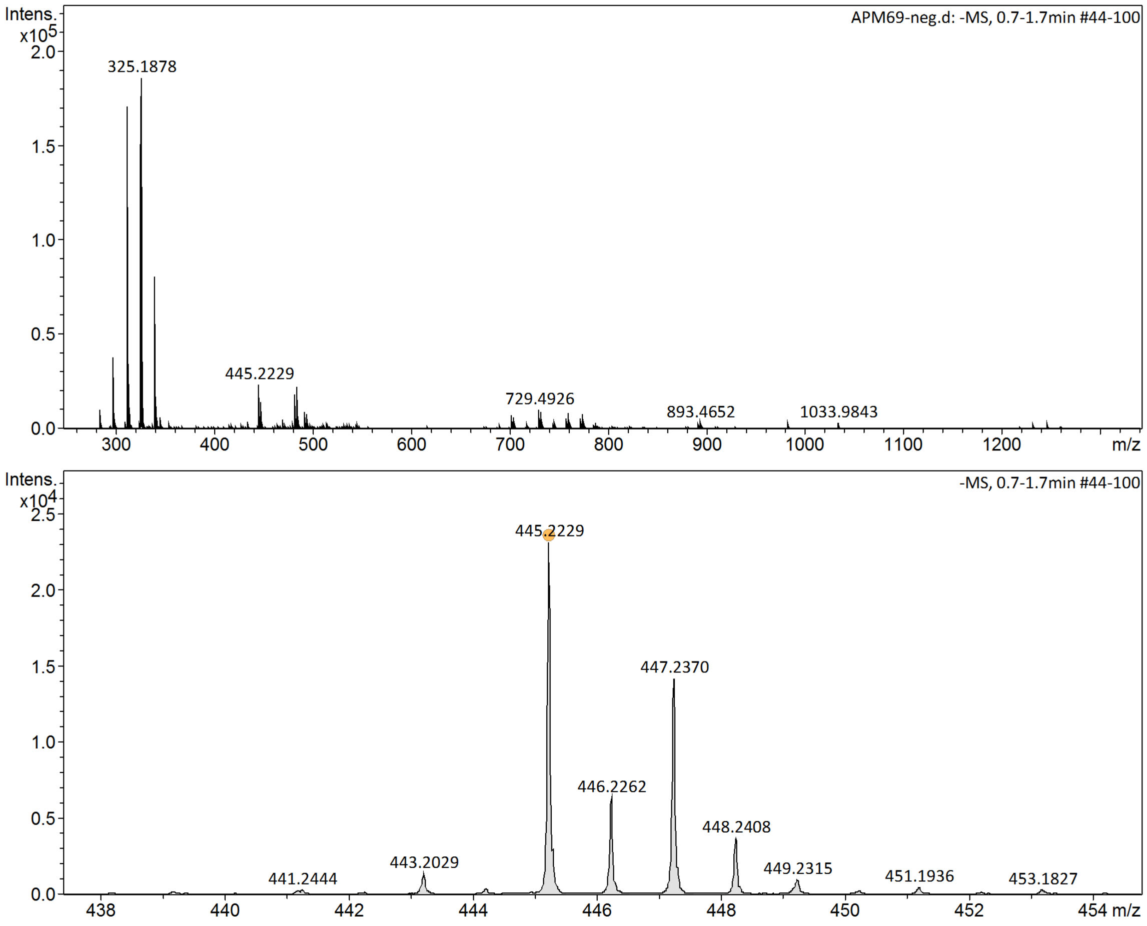

Meas. m/z	#	Ion Formula	m/z	err [ppm]	mSigma	# mSigma	Score	rdb	e¯ Conf	N-Rule
445.2229	1	C25H33O7	445.2232	0.7	291.4	1	100.00	9.5	even	ok

3. ESI HRMS of the sample **1d**/**1e** (2/1) positive mode.

Analysis Info

Analysis Name	D:\Data\Williams\APM69-pos.d	Acquisition Date	9/8/2016 4:54:41 PM
Method	tune_wide_pos_June 2016.m	Operator	BDAL@DE
Sample Name		Instrument	micrOTOF-Q 228888.00070
Comment			

Acquisition Parameter

Source Type	ESI	Ion Polarity	Positive	Set Nebulizer	0.4 Bar
Focus	Not active	Set Capillary	4500 V	Set Dry Heater	180 °C
Scan Begin	50 m/z	Set End Plate Offset	-500 V	Set Dry Gas	4.0 l/min
Scan End	3500 m/z	Set Collision Cell RF	550.0 Vpp	Set Divert Valve	Source

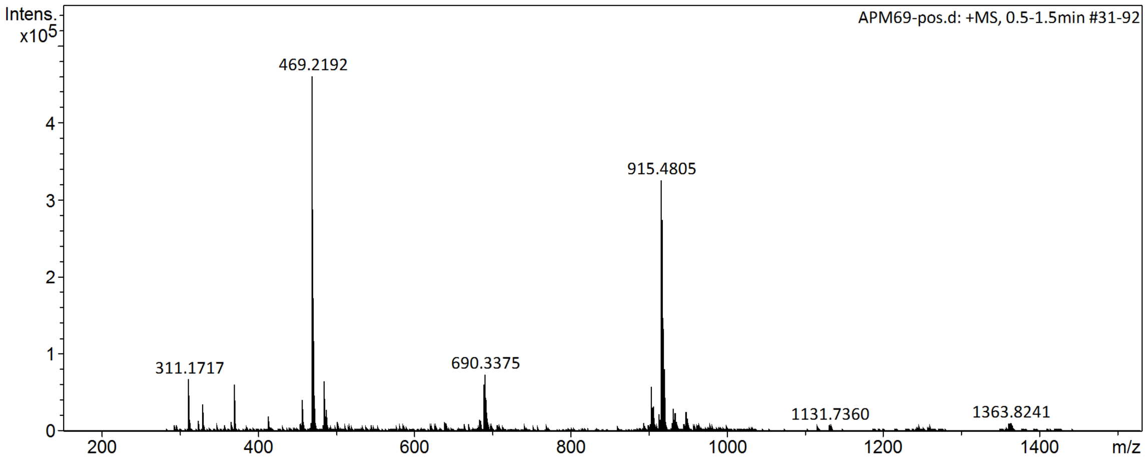

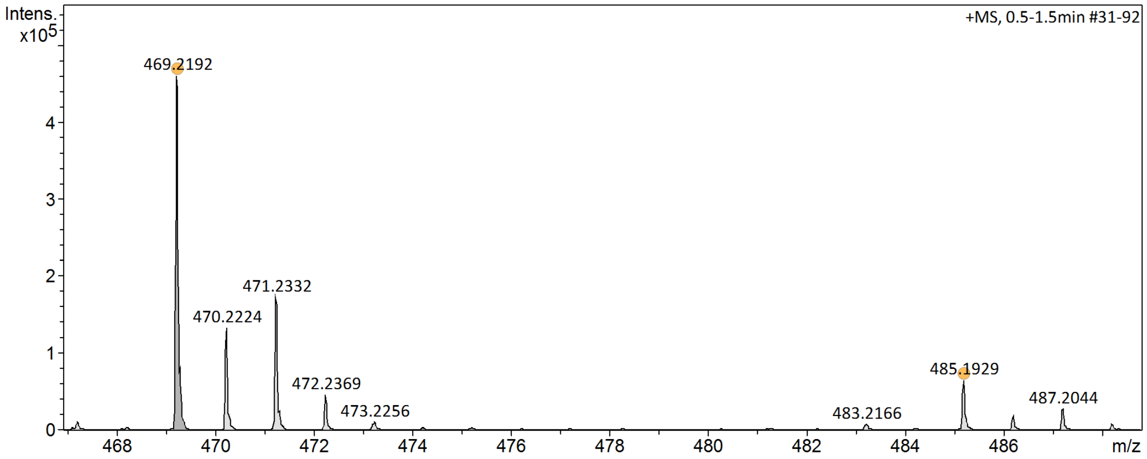

Meas. m/z	#	Ion Formula	m/z	err [ppm]	mSigma	# mSigma	Score	rdb	e¯ Conf	N-Rule
469.2192	1	C25H34NaO7	469.2197	1.1	166.9	1	100.00	8.5	even	ok
485.1929	1	C25H34KO7	485.1936	1.5	63.3	1	100.00	8.5	even	ok

4. Tabulated NMR data for **1d** compound recorded in $CDCl_3$ at 176/700 MHz.

No.	^{13}C	^{1}H	Multiplicity (*J* in Hz)
1	160.28	7.56	s
2	133.41		
3	208.80		
4	73.49		
5a	38.74	2.44	m
5b	38.74	2.54	m
6	140.74		
7	129.29	5.63	m
8	38.99	3.09	m
9	79.17		
10	56.77	3.17	br d (2.54)
11	43.50	2.15	m
12	87.36	4.84	d (9.81)
13	60.83		
14	35.19	0.90	m
15	27.69		
16	22.27	1.16	s
17	17.03	1.01	s
18	16.08	1.03	u
19	10.14	1.77	dd (2.91, 1.27)
20a	67.97	3.98	m
20b	67.97	4.03	m
1'	170.51		
2'	128.13		
3'	138.66	6.85	qd (7.08, 1.27)
4'	14.51	1.78	m
5'	12.05	1.80	d (1.09)
9-OH		4.93	br s

5. ^{1}H NMR spectrum and assignments for **1d** recorded in $CDCl_3$. In expansions the signals of the minor components are observed.

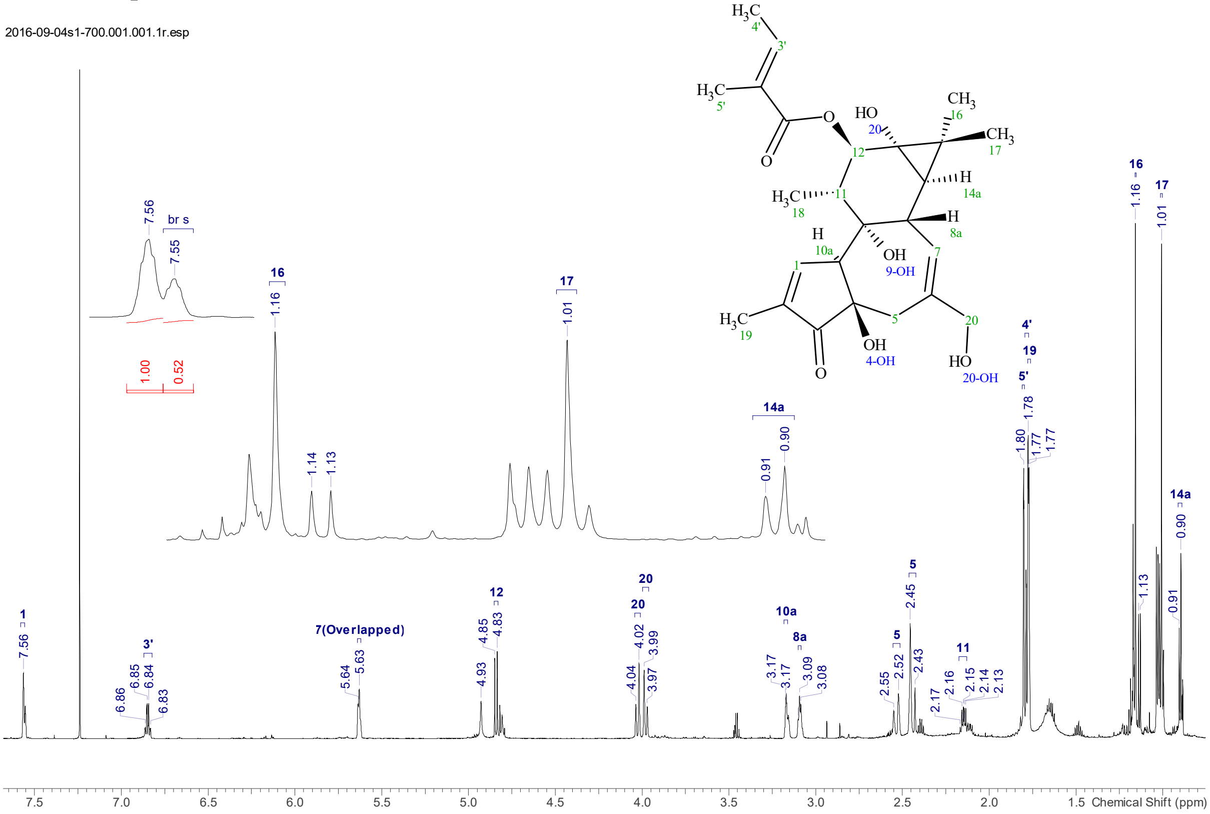

6. ^{13}C NMR spectrum and assignments for **1d** recorded in $CDCl_3$. In expansions the signals of the minor components are observed.

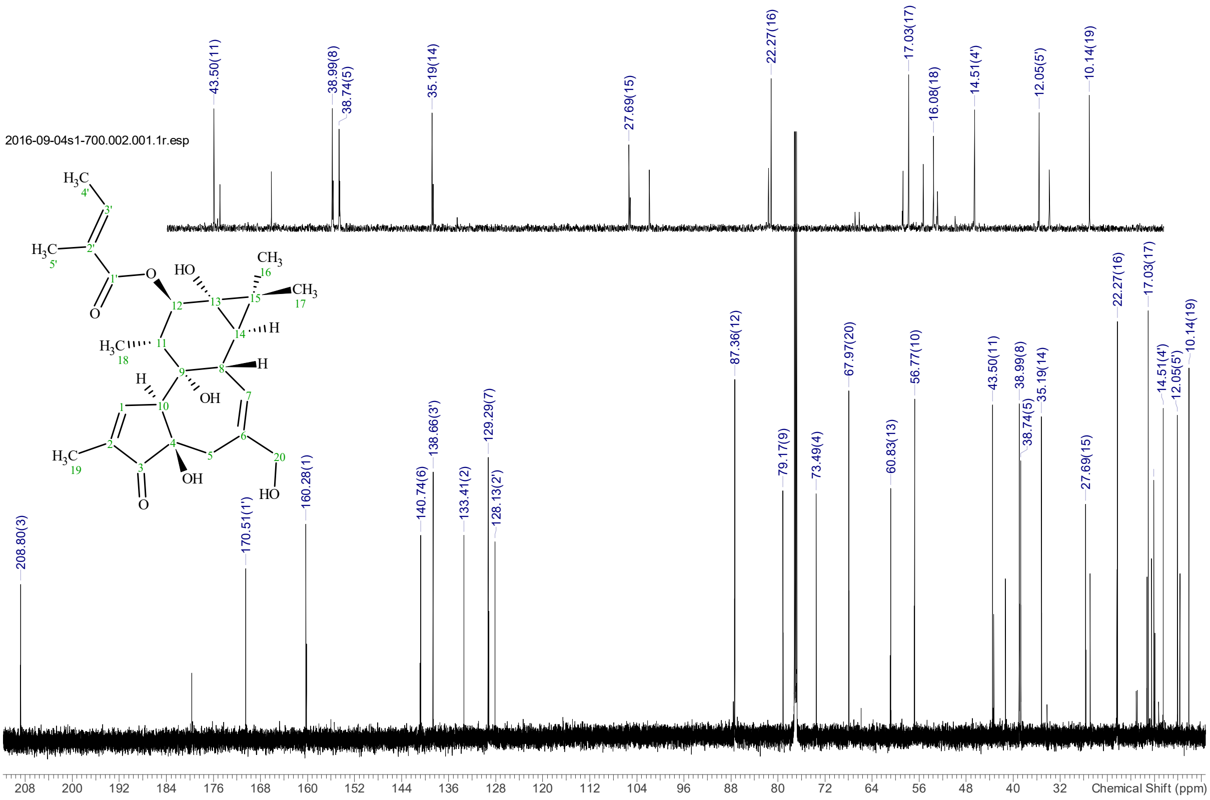

7. DEPT spectrum for **1d** recorded in $CDCl_3$.

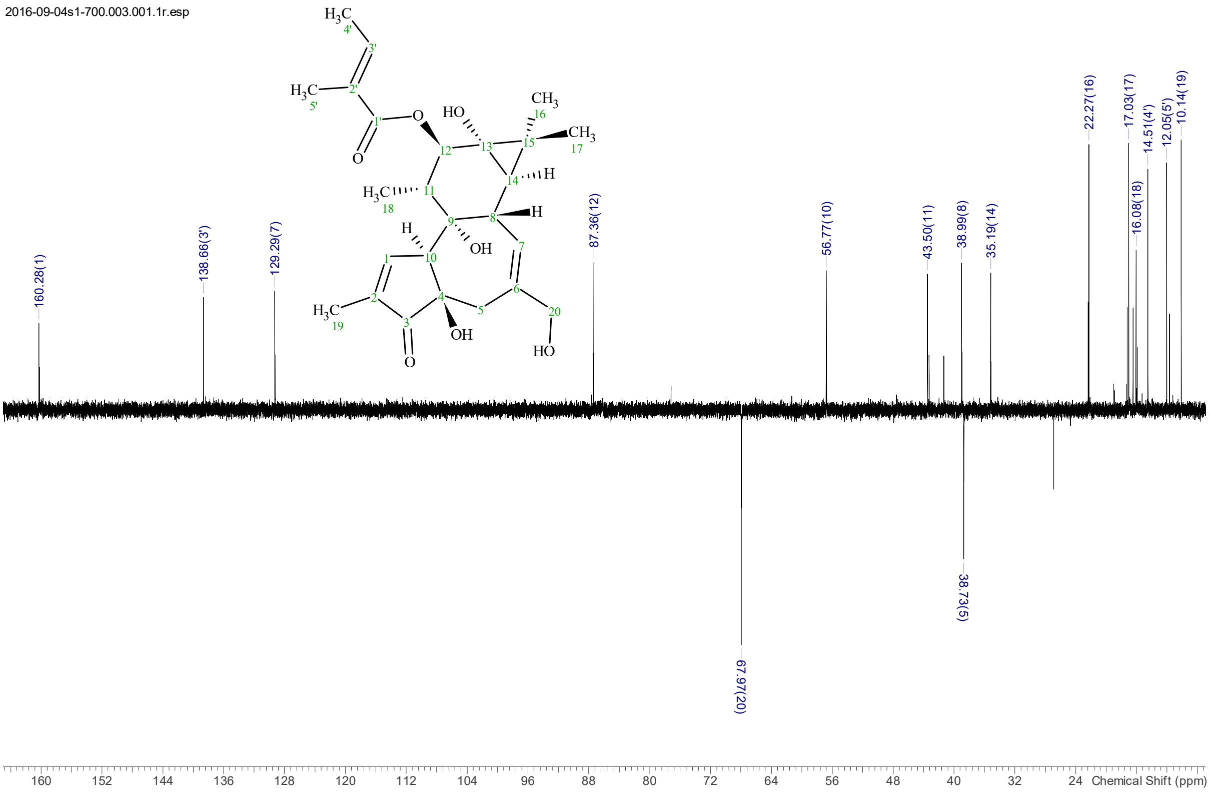

8. COSY spectrum for **1d** recorded in $CDCl_3$.

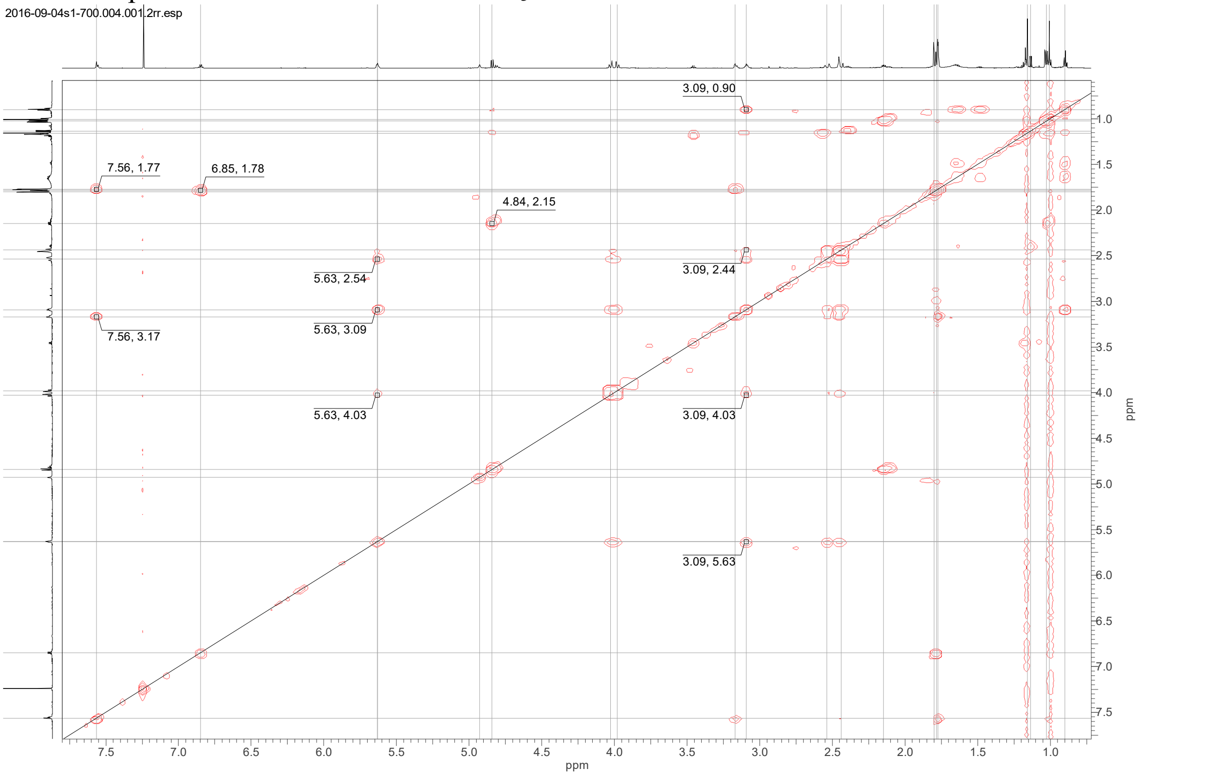

9. HSQC edit spectrum for **1d** recorded in $CDCl_3$.

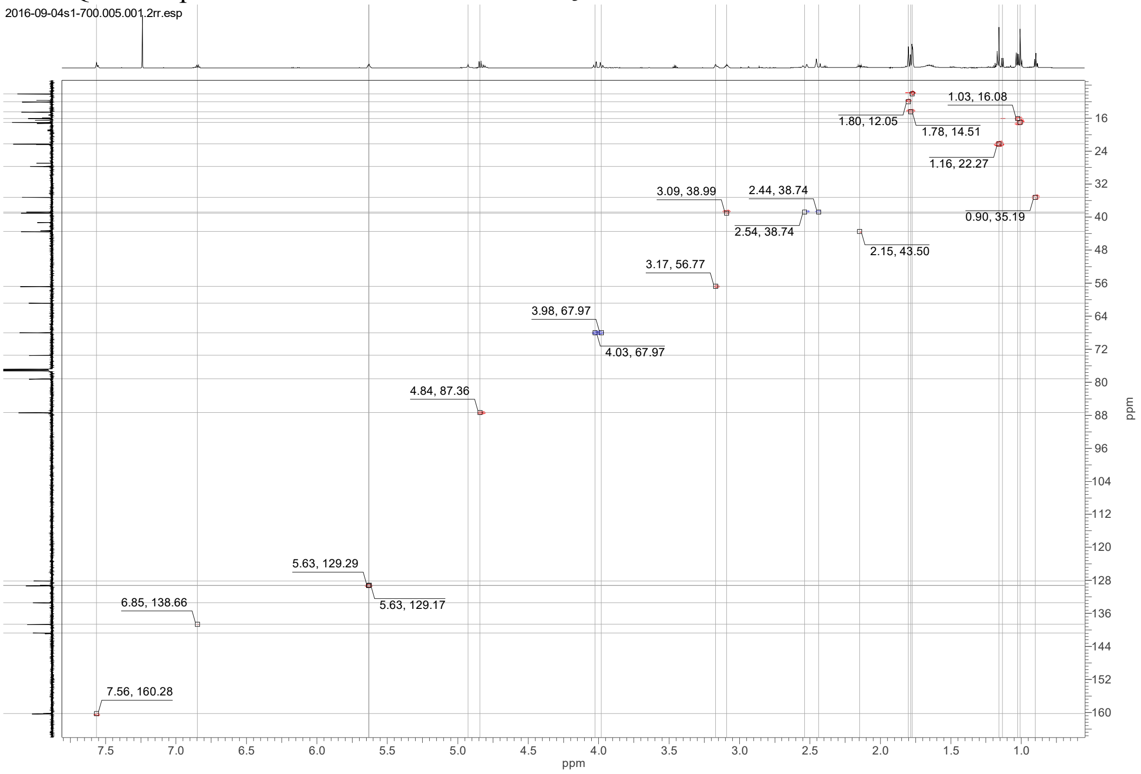

10. HSQC spectrum for **1d** recorded in $CDCl_3$, 0.7-2.0×5-45 ppm expansion.

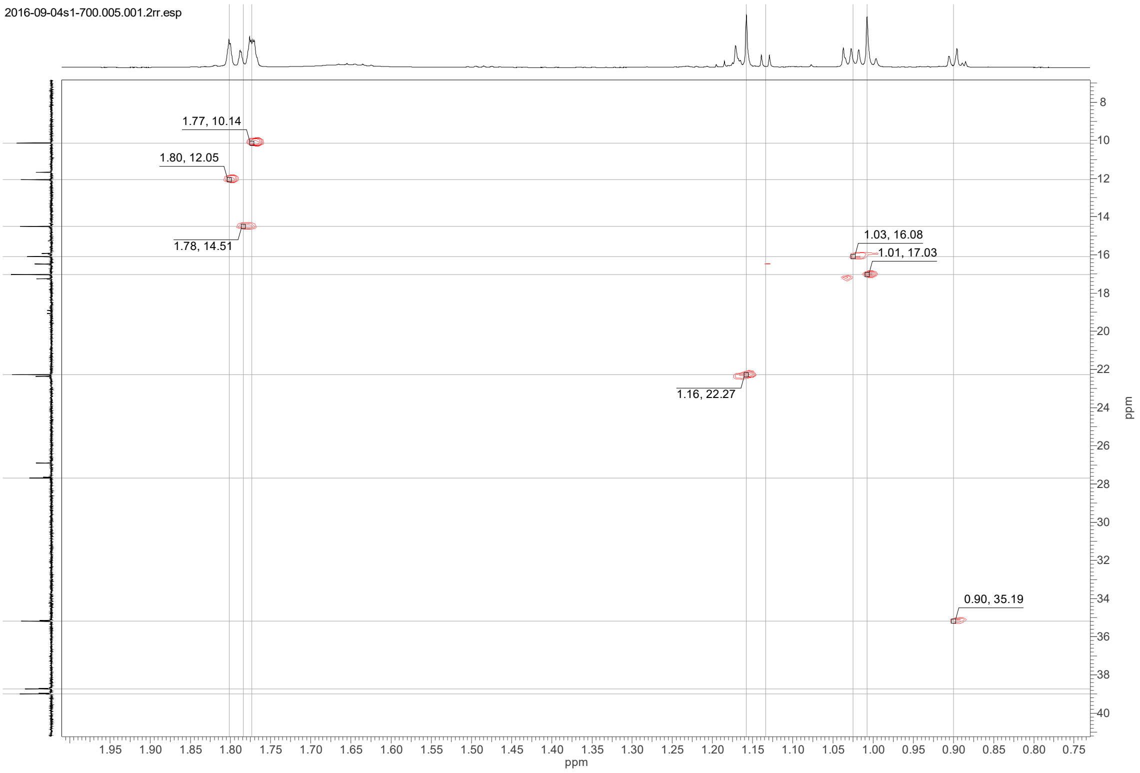

11. HMBC spectrum for **1d** recorded in $CDCl_3$.

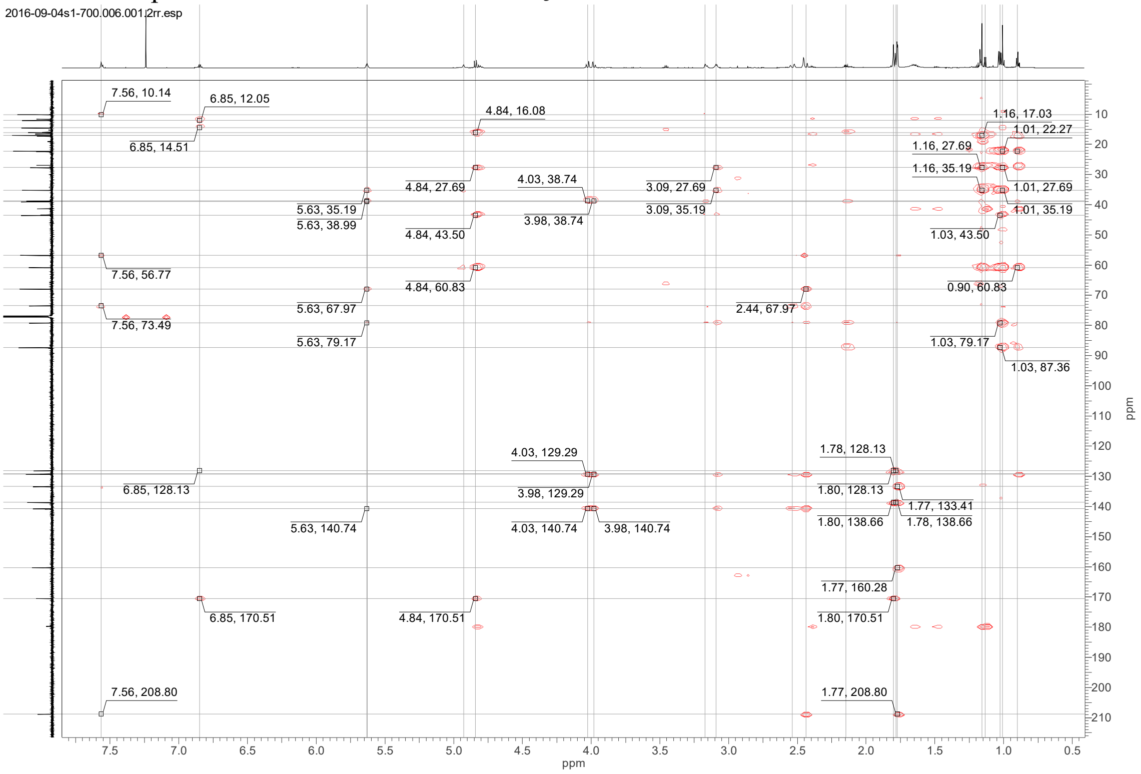

12. HMBC spectrum for **1d** recorded in $CDCl_3$, 1.40-7.40×2-218 ppm.

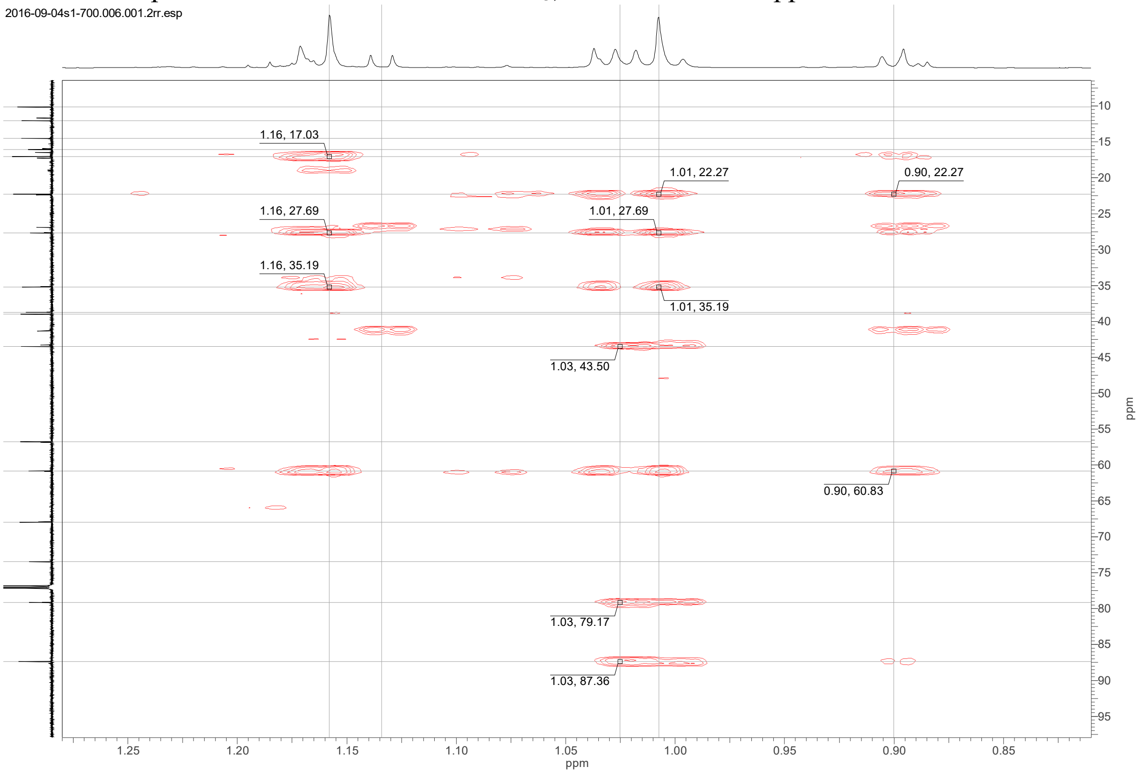

13. NOESY spectrum for **1d** recorded in $CDCl_3$.

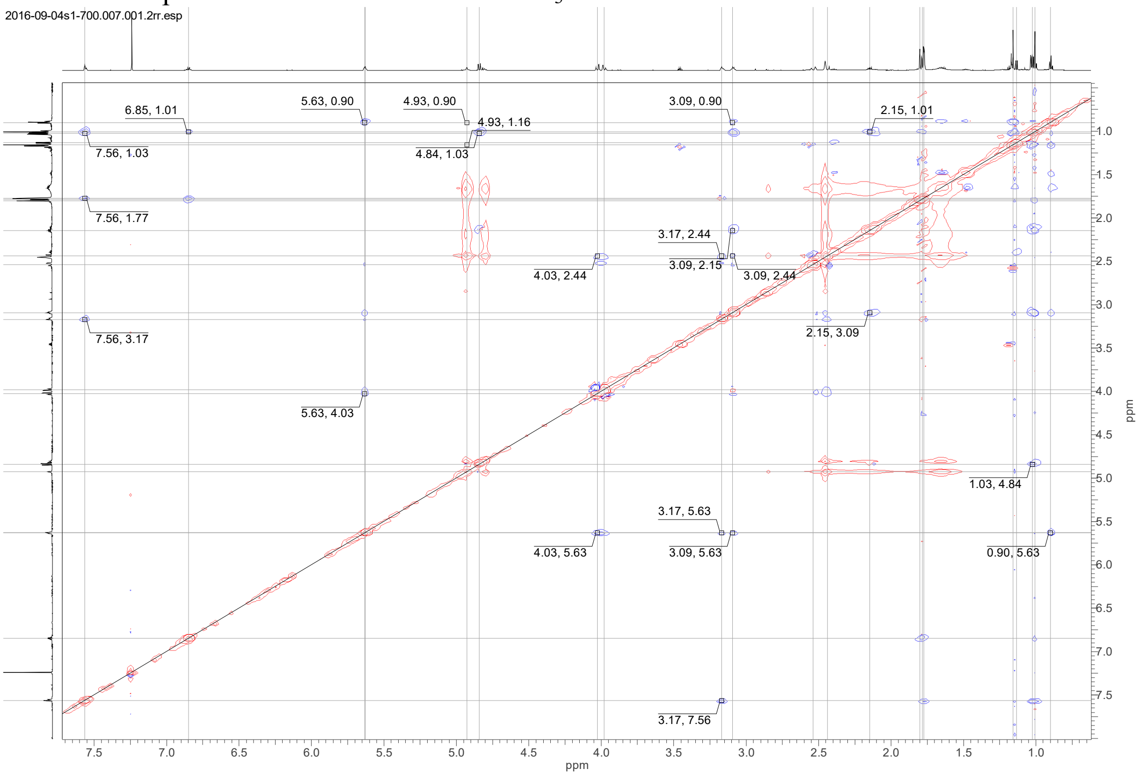

14. Tabulated NMR data for **1e** compound recorded in $CDCl_3$ at 176/700 MHz.

**No.**	**^{13}C**	**^{1}H**	**Multiplicity (*J* in Hz)**
1	160.18	7.55	br s
2	133.42		
3	208.77		
4	73.47		
5a	38.70	2.44	br d (16.35)
5b	38.70	2.54	d (19.07)
6	140.84		
7	129.17	5.63	br d (5.09)
8a	38.96	3.09	br d (5.63)
9	79.12		
10a	56.82	3.16	br d (3.27)
11	43.28	2.11	dq (9.99, 6.72)
12	87.41	4.83	d (9.99)
13	60.89		
14a	35.14	0.89	d (7.63)
15	27.63		
16	22.37	1.17	s
17	17.24	1.04	s
18	15.92	1.00	d (6.50)
19	10.13	1.77	dd (2.91, 1.27)
20a	67.92	3.98	d (12.90)
20b	67.92	4.03	d (12.90)
1'	179.68		
2'	41.31	2.39	td (13.94, 6.99)
3'	26.90	1.65	m
3'	26.90	1.48	tt (13.74, 7.43)
4'	11.67	0.90	t (5.99)
5'	16.47	1.13	d (6.90)

15. ^{1}H NMR spectrum and assignments for **1e** recorded in $CDCl_3$, expansions are shown for selected regions to present the signals of the minor component.

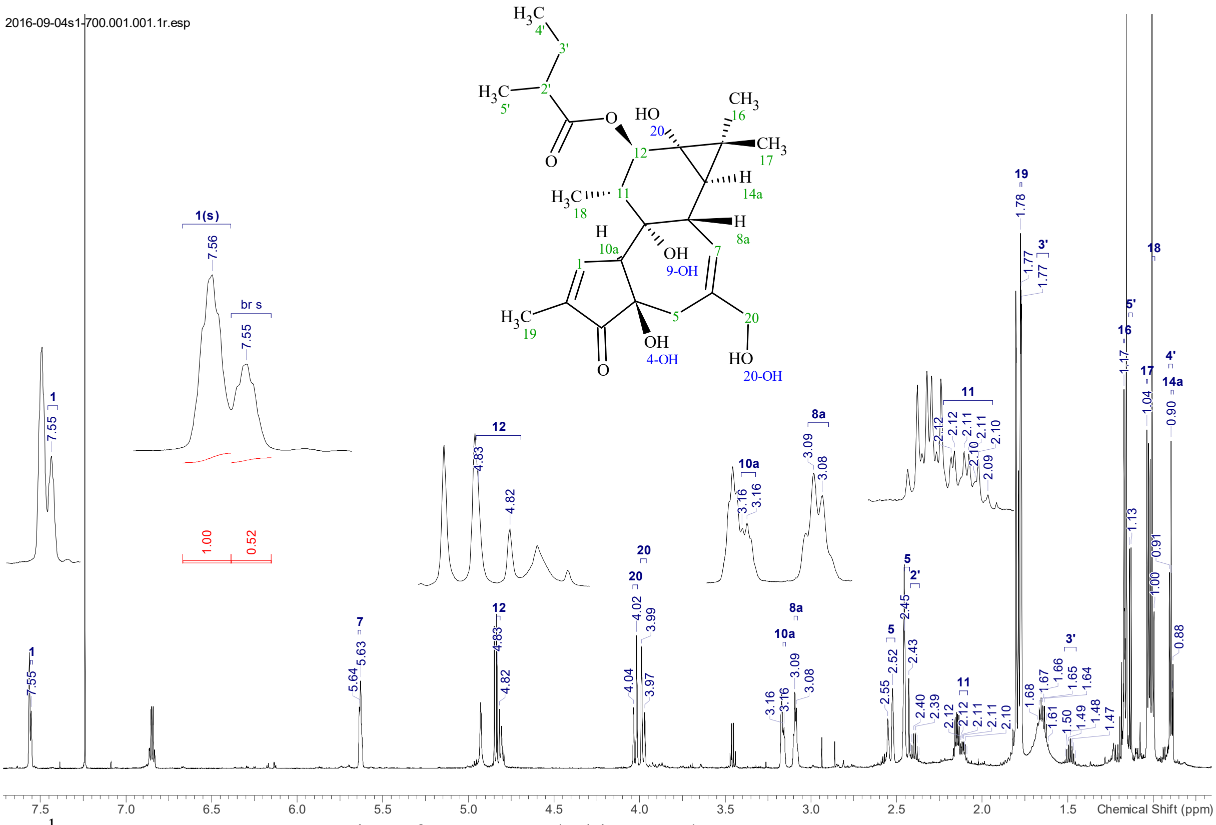

16. ^{1}H NMR spectrum expansions for **1e** recorded in $CDCl_3$.

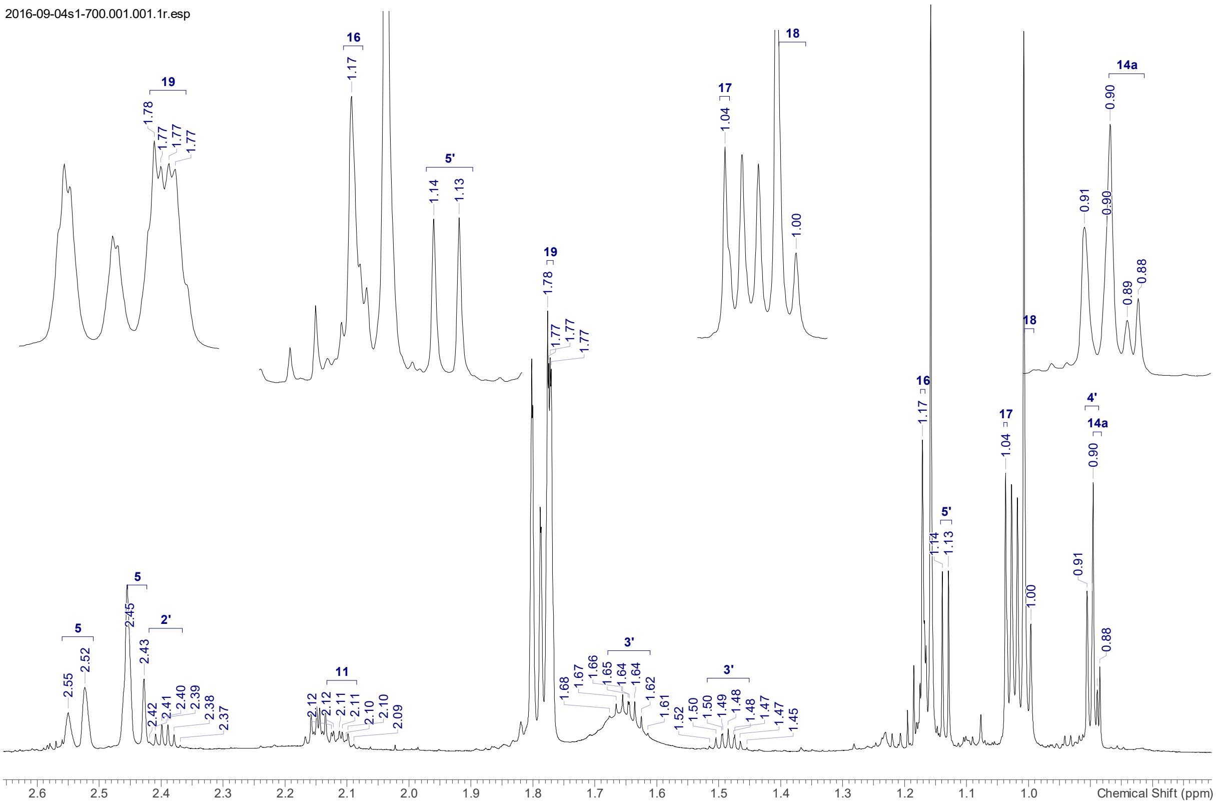

17. ^{13}C NMR spectrum and assignments for **1e** recorded in $CDCl_3$, expansions are shown for the selected carbons.

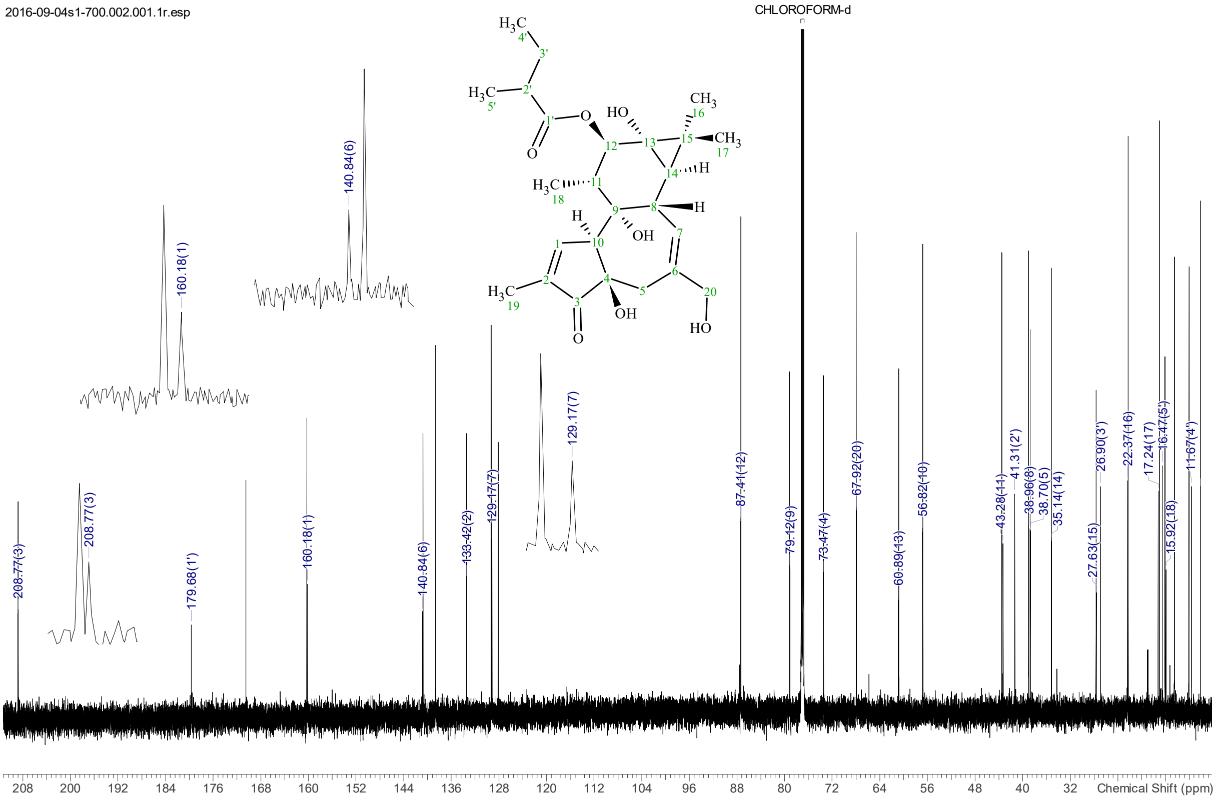

18. ^{13}C NMR spectrum expansions for **1e** recorded in $CDCl_3$.

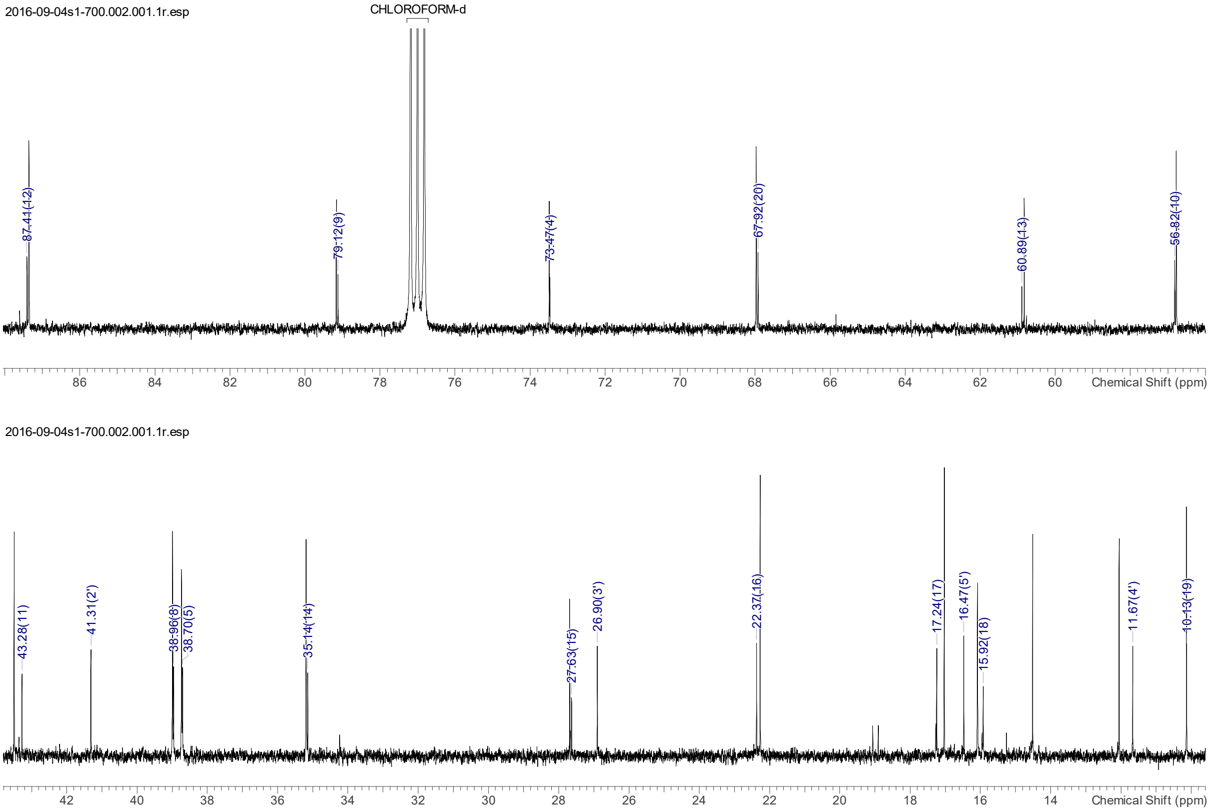

19 DEPT spectrum and assignments for **1e** recorded in $CDCl_3$.

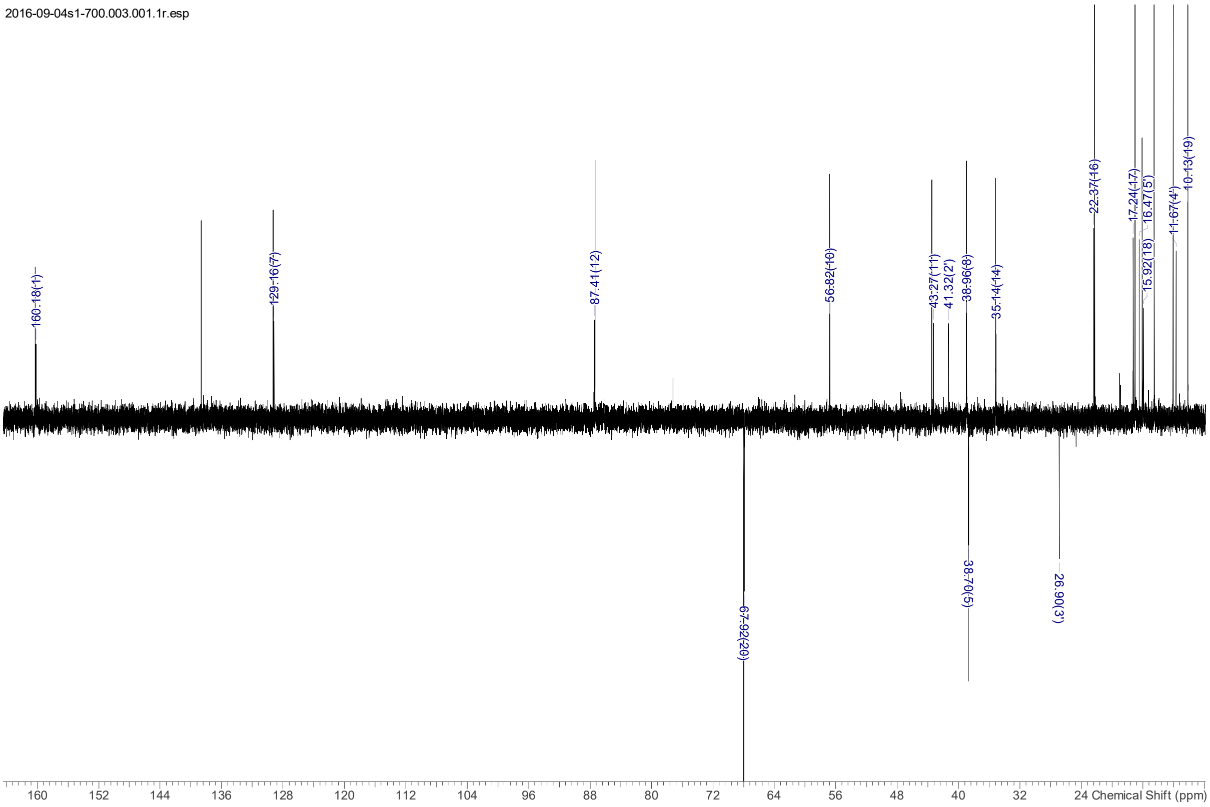

20. COSY spectrum for **1e** recorded in $CDCl_3$, peak picking was performed for the minor component.

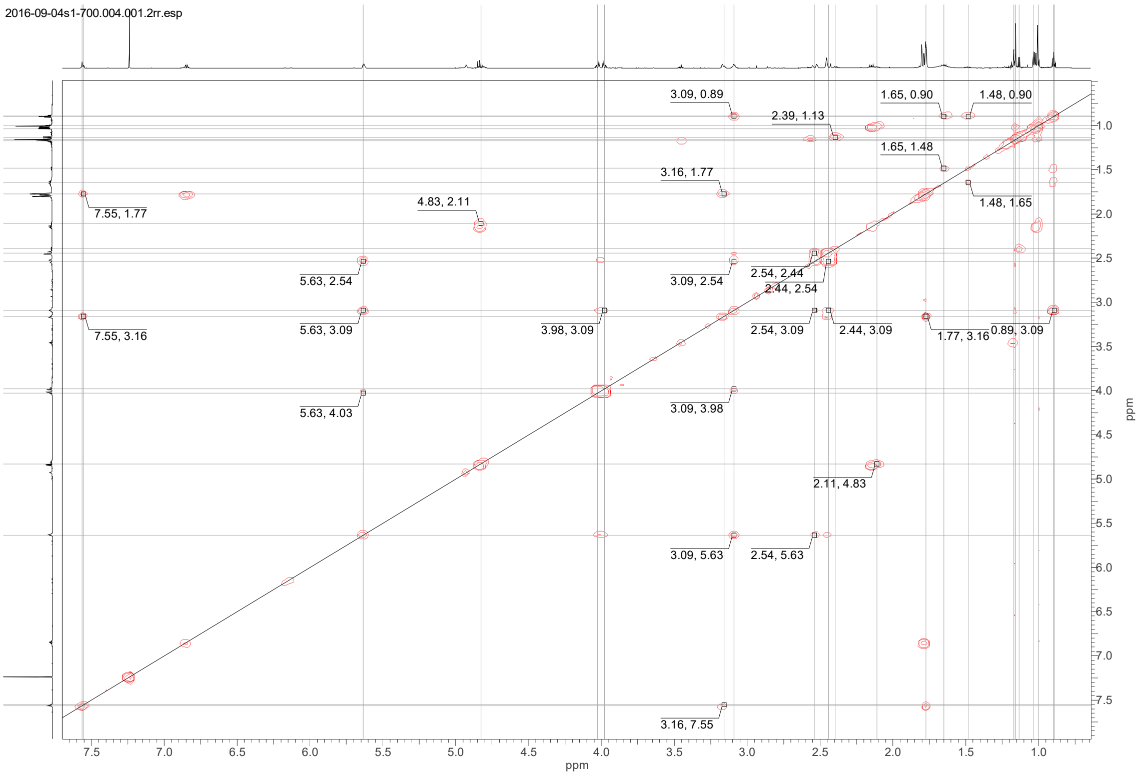

21. HSQC edit spectrum for **1e** recorded in $CDCl_3$, peak picking was performed for the minor component.

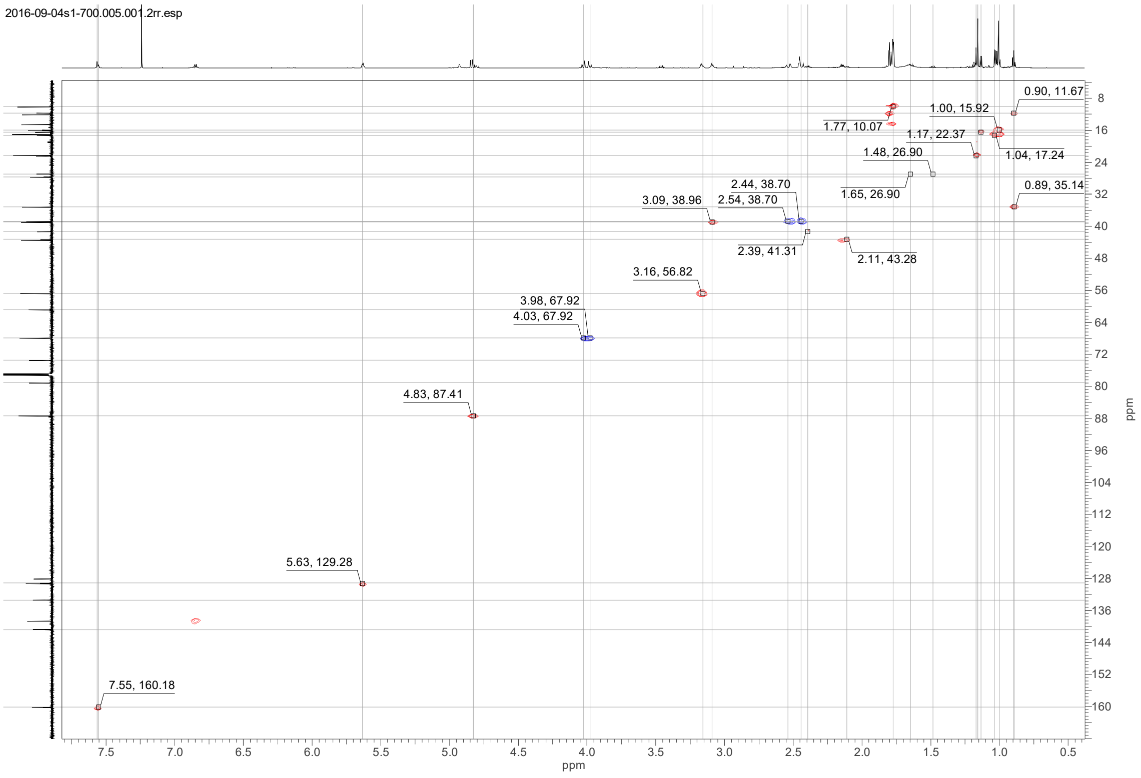

22. HSQC spectrum for **1e** recorded in $CDCl_3$, 0.7-3.1×5-58 ppm expansion, peak picking was performed for the minor component.

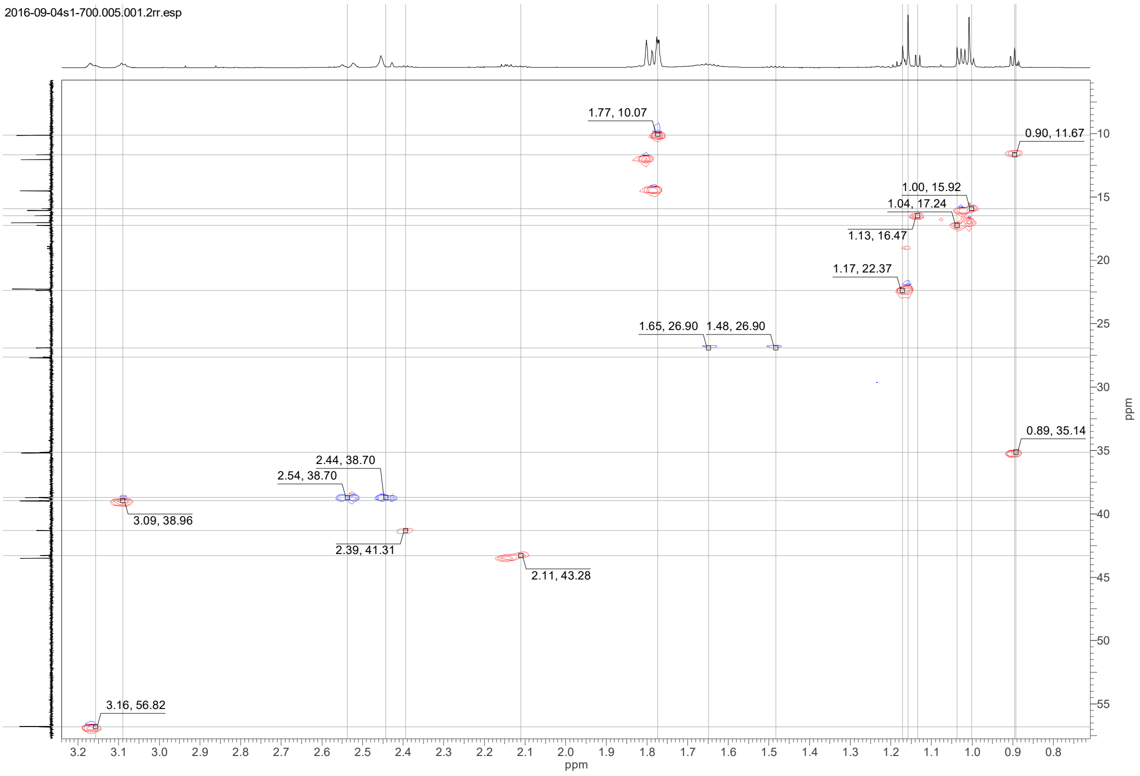

23. HMBC spectrum for **1e** recorded in $CDCl_3$, peak picking was performed for the minor component.

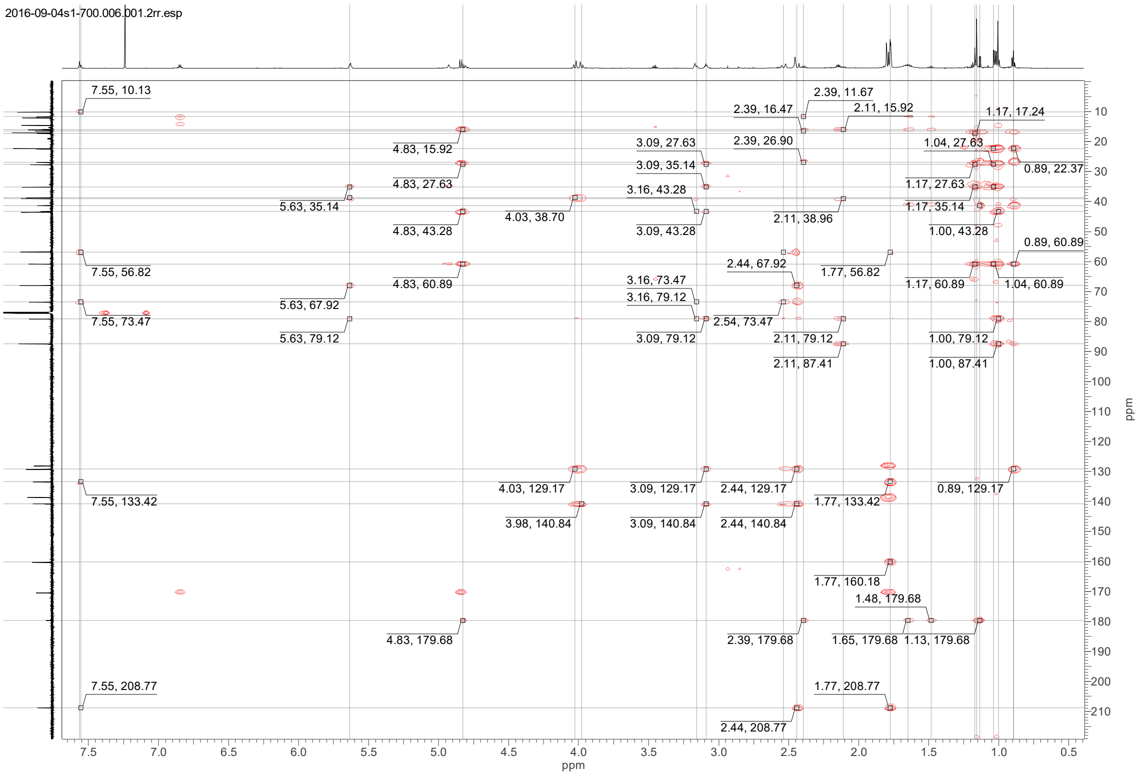

24. HMBC spectrum for **1e** recorded in $CDCl_3$, 0.7-2.6×5-95 ppm expansion, peak picking was performed for the minor component.

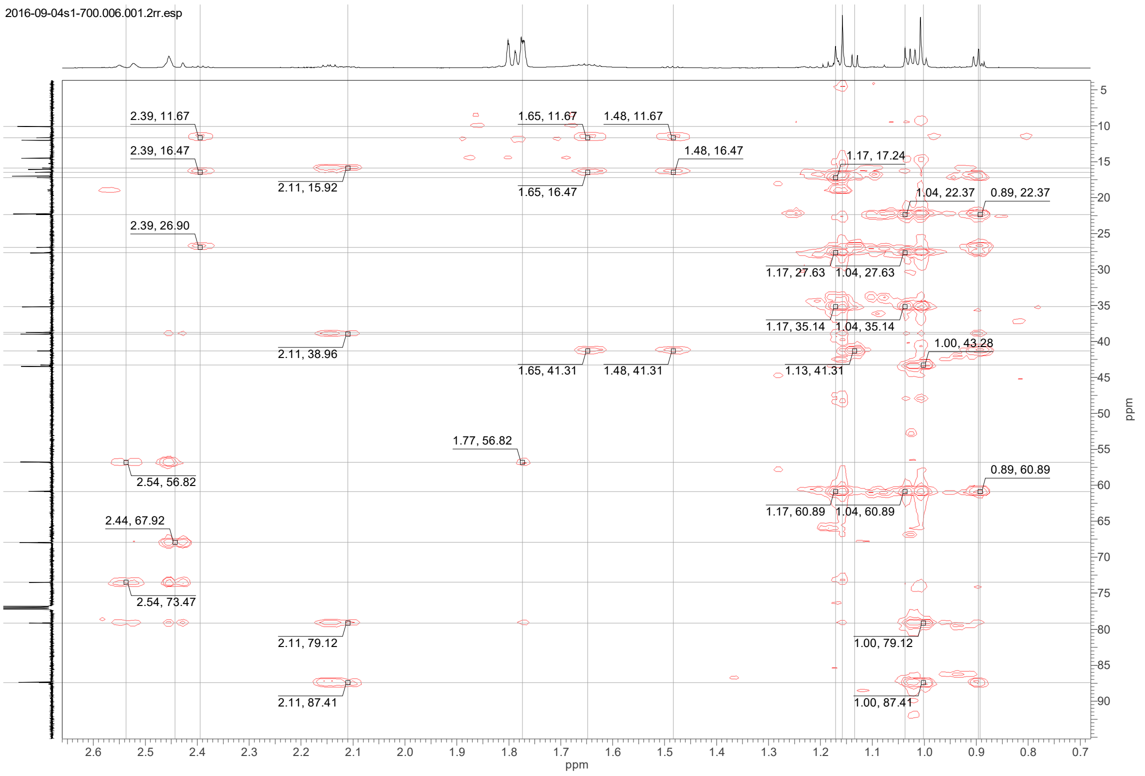

25. NOESY spectrum for **1e** recorded in $CDCl_3$, peak picking was performed for the minor component.

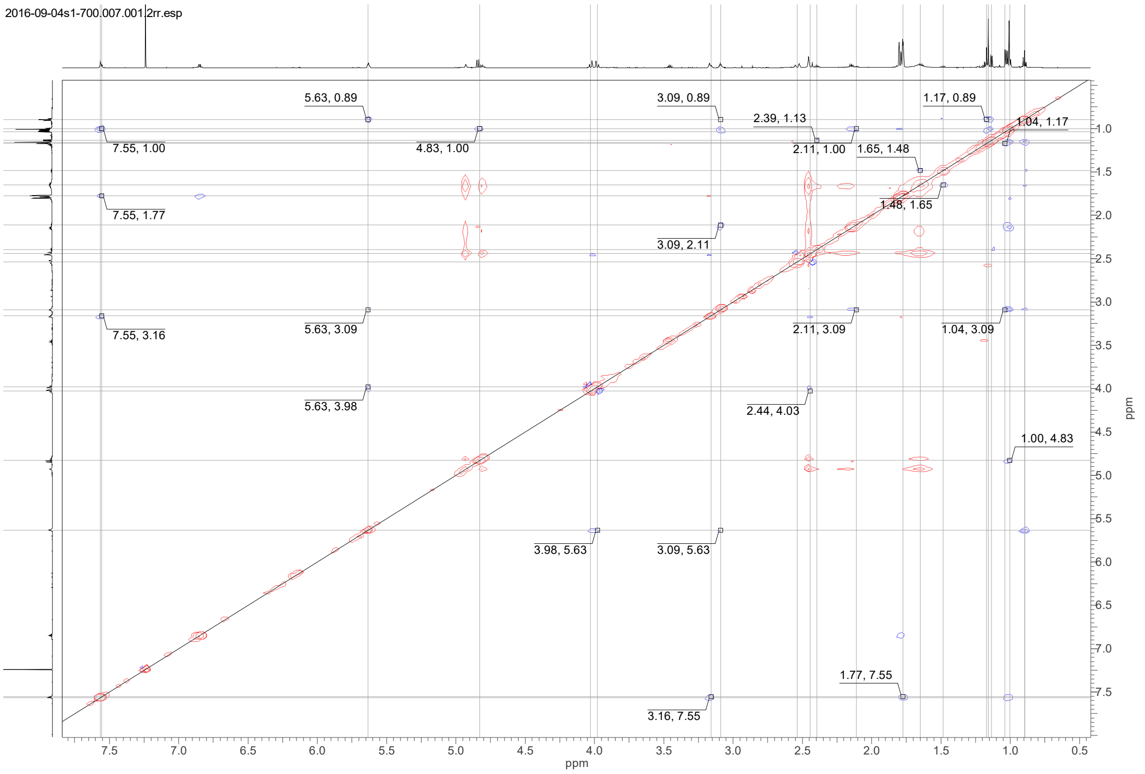

26. NOESY spectrum for **1e** recorded in $CDCl_3$, 0.5-5.0×0.7-3.3 ppm expansion, peak picking was performed for the minor component.

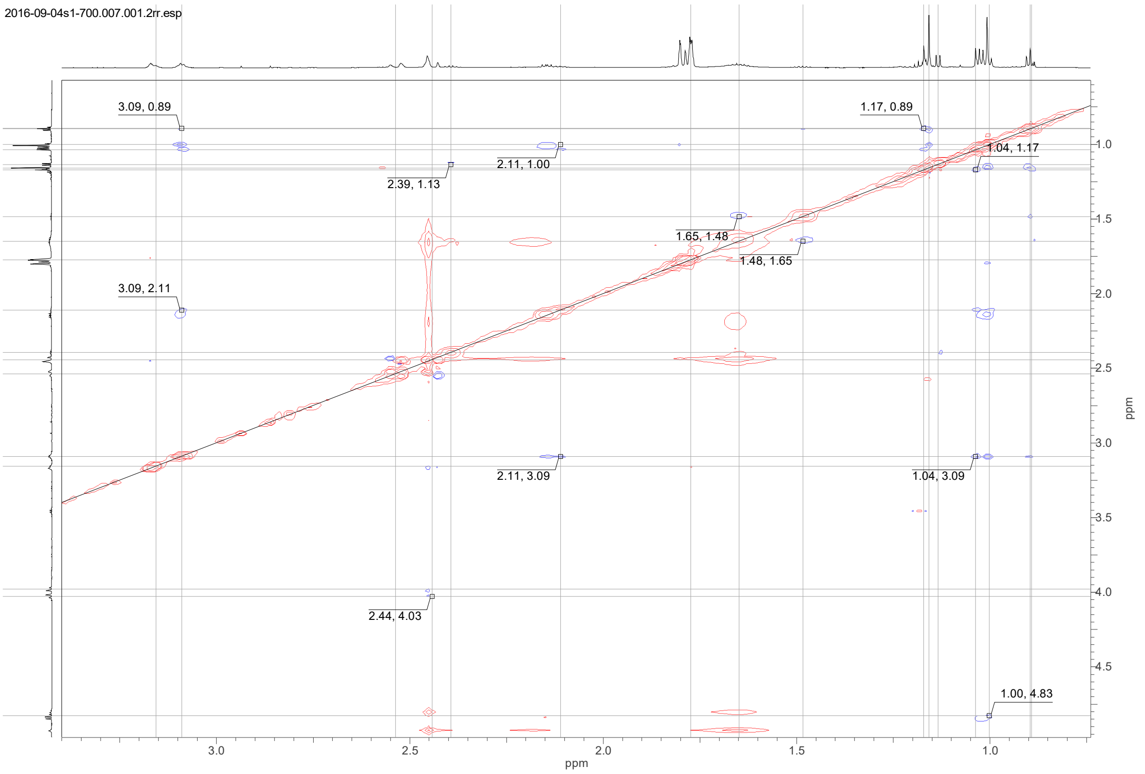

Supplement: File 1 — ESI-HRMS and 1H and 13C NMR spectra of compounds 1d and 1e. [file Beilstein_J_Org_Chem-13-1361-s001.pdf]
